# The disruptive influence of the Ala218Val variant on the ENG protein

**DOI:** 10.17912/micropub.biology.001350

**Published:** 2025-02-26

**Authors:** Jared Truitt, Cynthia L Stenger, Luke Terwilliger, Michele Morris

**Affiliations:** 1 CSIS, University of North Alabama, Florence, Alabama, United States; 2 Mathematics, University of North Alabama, Florence, Alabama, United States; 3 HudsonAlpha Institute for Biotechnology, Huntsville, Alabama, United States

## Abstract

Hereditary Hemorrhagic Telangiectasia (HHT) is an autosomal dominant disease that interferes with the formation of arteries. The *ENG* gene encodes for the protein endoglin which is used to properly develop and remodel arteries. The removal of endoglin forms telangiectasias that cause bleeding from the nose and vital organs. This study investigates the impact of one of the many variants of uncertain significance of ENG associated with HHT. The missense swap of alanine for valine at position 218 (Ala218Val) was characterized through computational metrics from in silico pathogenicity prediction tools, conservation analysis, and molecular dynamics simulation (MDS). The structural residue is highly conserved over multiple species and buried. The missense swap resulted in a difference in movement from the wild type according to MDS in a simulated aqueous environment. Therefore, it is predicted to be likely pathogenic.

**Figure 1. Characterization of ENG Variant ALA218Val:**
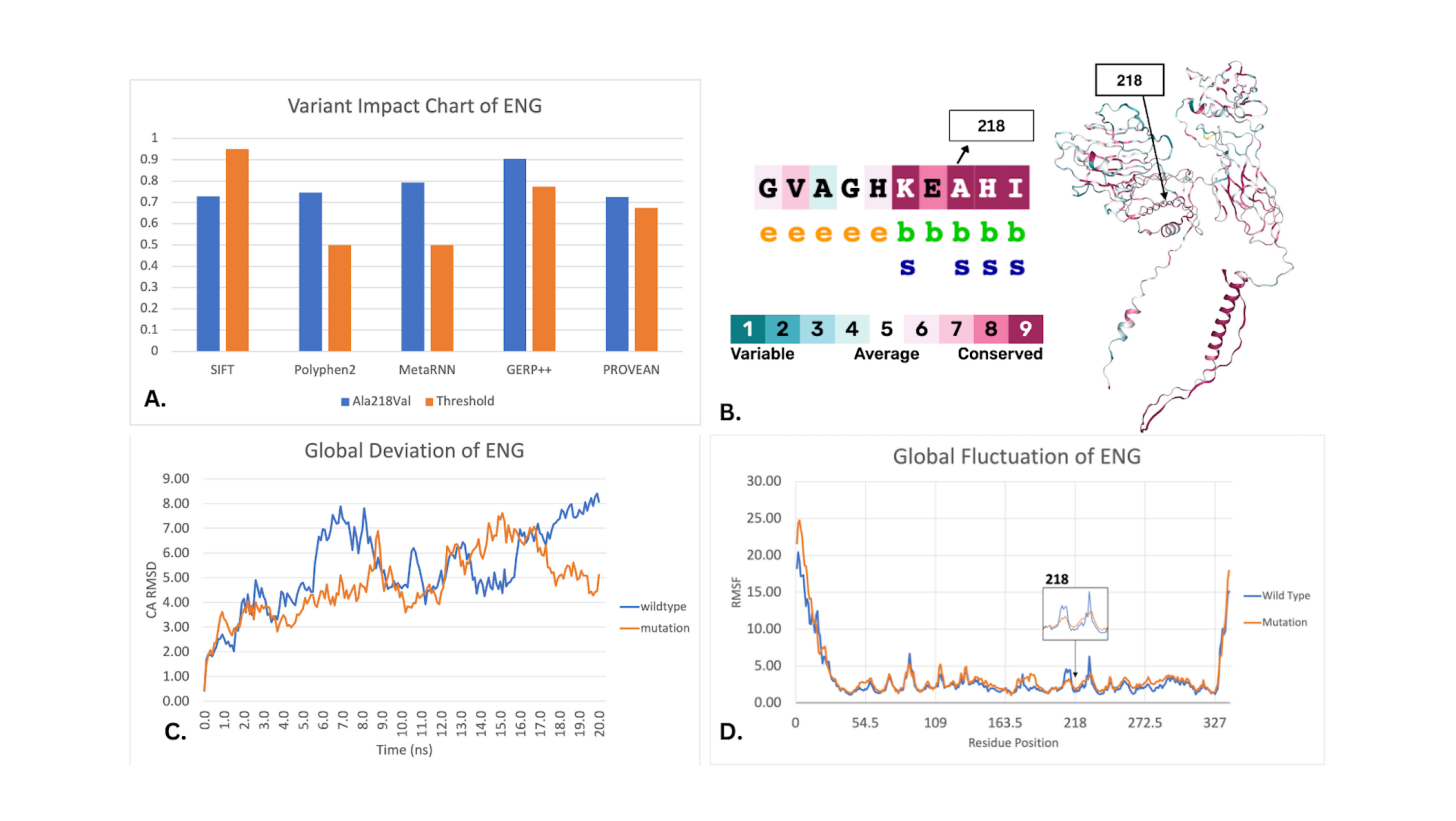
A) Comparison of prediction for Ala218Val to the pathogenicity threshold for each computational tool. All resulting scores except SIFT were above the threshold (Abzhubei et al., 2013; Llorca et al., 2007; Ng & Henikoff, 2001). B) Conservation ranking of the Ala218Val residue based on ConSurf, predicted the variant to be a structural residue - indicated by the s - that is highly conserved and buried - indicated by the b (Landau et al., 2005). C) Molecular dynamics simulations run on the native type and on the variant Ala218Val show the root mean squared deviations (RMSD), calculated over 20 nanoseconds in a simulated aqueous environment. D) The RMSF of the wild-type and mutation of Ala218Val show the fluctuation by amino acid position. An arrow points to position 218 of the protein. A visual difference is observed (Land & Humble, 2018).

## Description

Hereditary Hemorrhagic Telangiectasia (HHT) is an autosomal dominant disease caused by mutations in the human endoglin gene ( *ENG* ), which encodes for the protein by the same name (Adzhubei et al., 2013) . HHT is known to cause telangiectasias, or small dilated blood vessels, on the surface of the skin and on vital organs. These telangiectasias can cause disruptive bleeding and sometimes death. Misdiagnosis or delay in diagnosis are common, especially in infants and young patients under 21 years of age. Often, they do not realize they have HHT until they experience a life-threatening condition. Endoglin is a transmembrane protein that is expressed on the surface of endothelial cells. It is required for angiogenesis which is the formation of blood vessels (Adzhubei et al., 2013) . The variant that results from a swap of alanine for valine at position 218 in *ENG* has been associated with HHT in a clinical submission but has not been classified as significant. The variant was selected due to its proximity to known pathogenic variants, together with its predicted deleterious CADD score (Rentzsch et al., 2021) . The swap from alanine to valine is a nonpolar hydrophobic change and it occurs in a highly conserved region that is buried within the structure of the gene. Nonpolar, buried hydrophobic residues can interfere with hydrophobic core packing and are common among known pathogenic variants in ENG (Gallione et al., 2000; Saito et al., 2017) .

Multiple in silico analysis prediction tools, including SIFT, PolyPhen-2, MetaRNN, Provean, and GERP++ provided insights into the variant's impact on protein function, evolutionary conservation, and genomic context ( Figure 1 A ). The Ala218Val variant is compared to the relative pathogenicity threshold of each in silico analysis prediction score, showing all scores above the threshold except for one, SIFT, which does not consider the effect a mutation can have on the protein's structure (Landau et al., 2005) . This could be the reason SIFT does not predict Ala218Val, a deeply buried and structural variant, to be pathogenic.

Conservation analysis via ConSurf showed the variant's evolutionary significance and its potential influence on the structural integrity of the gene. Aligning multiple sequences showed position 128 was highly conserved and buried. This supports the importance of Ala218Val for the structural integrity of the gene ( Figure 1 B) (Land & Humble, 2018; Landau et al., 2005) .

Finally, molecular dynamics was used to simulate the movement of the native and the variant in an aqueous environment over 20 ns. The simulations visually demonstrated significant structural impacts, as evidenced by deviations and fluctuations between the wild-type and mutated gene ( Figure 1 C-D ). Comparing the movement differential over time and by amino acid position. There is a statistically significant difference between the wild-type RMSD and the mutation RMSD, implying that the amino acid swap of alanine to valine could have a structural or functional impact of other regions in the gene. It should be noted that our dynamics simulation did not code for the transmembrane portion of this protein. Also, the 20ns timeline was arbitrarily chosen based on previous research.

These collective findings underscore the potential pathogenicity of the Ala218Val variant, suggesting a disruptive influence on the structural integrity of the ENG gene, thereby contributing valuable insights into its role in Hereditary Hemorrhagic Telangiectasia .

## Methods

Genetic variations associated with HHT are predominately attributed to ENG. Therefore, ENG was chosen for study due to its high occurence in clinical submissions related to HHT. A variant of unknown significance (VUS) from ENG resulting from a missense swap of alanine to valine at position 218 was chosen for study due to its association with HHT in clinical submission, its proximity to known pathogenic variants, and its predicted deleterious CADD score (Rentzsch et al., 2021) . Multiple normalized scores from various pathogenicity predictors (Ng & Henikoff, 2001; Adzhubei et al., 2013; Li et al., 2022; Choi & Chan, 2015; Davydov et al., 2010) and the ConSurf server (Landau et al., 2005) were used to assess the functional impact of Ala218Val, focusing on its potential influence on protein function and evolutionary conservation. To further investigate the structural impact, molecular dynamics simulations were performed on both the native and Ala218Val ENG variants in an aqueous environment over 20 nanoseconds. Through these simulations, root mean square deviation (RMSD) values were calculated over time and across amino acid positions to visualize structural changes. These methods support further experimental studies to validate these findings in the context of HHT pathology.
